# Differential Effects of Rutin and Its Aglycone Quercetin on Cytotoxicity and Chemosensitization of HCT 116 Colon Cancer Cells to Anticancer Drugs 5-Fluorouracil and Doxorubicin

**DOI:** 10.3390/biology14050527

**Published:** 2025-05-09

**Authors:** Iva Suman, Alberta Jezidžić, Dorotea Dobrić, Robert Domitrović

**Affiliations:** 1 Department of Medical Chemistry, Biochemistry and Clinical Chemistry, Faculty of Medicine, University of Rijeka, Braće Branchetta 20, 51000 Rijeka, Croatia; iva.potocnjak@uniri.hr (I.S.);; 2 Department of Biotechnology, University of Rijeka, Radmile Matejčić 2, 51000 Rijeka, Croatia

**Keywords:** rutin, quercetin, flavonoids, 5-fluorouracil, doxorubicin, colon cancer, cytotoxicity, chemosensitization

## Abstract

Background: Rutin and quercetin are natural flavonoids with a variety of beneficial health effects, including anticancer activity. In the present study, we compared cytotoxicity and chemosensitization of human colon cancer HCT116 cells to anticancer drugs 5-fluorouracil (5-FU) and doxorubicin (DOX) by both compounds. Methods: The 2,3-bis(2-methoxy-4-nitro-5-sulfophenyl)-2H-tetrazolium-5-carboxanilide (XTT) test was used to determine cell viability. Western blot and immunofluorescence techniques were employed in the detection of expression of proteins involved in oxidative stress, apoptosis, and autophagy. Results: Quercetin treatment resulted in reduced cell viability compared to rutin at the same dose, suggesting greater cytotoxicity than rutin against HCT116 cells. Quercetin was also a better chemosensitizer of DOX than rutin, further reducing cell viability. However, rutin was a better chemosensitizer of 5-FU than quercetin. All treatments induced apoptosis, with rutin and DOX inducing intrinsic and 5-FU inducing extrinsic apoptotic cell death. Autophagy was induced in all treatments and played a pro-survival role, with the exception of DOX treatment. Different treatment regimens specifically modulated cancer cell signaling pathways involved in the regulation of oxidative stress, apoptosis, and autophagy. Conclusions: The results of the current study suggest that rutin and quercetin, although structural analogs, act as specific modulators of signaling pathways in cancer cells, differentially affecting cancer cell cytotoxicity and chemosensitization to anticancer drugs, based on the presence of a free hydroxyl group at the C-3 position of the flavonoid backbone at quercetin or rutinose in rutin.

## Simple Summary

Rutin and quercetin are natural flavonoids with anticancer activity. Both compounds were cytotoxic to HCT116 colon cancer cells and chemosensitized the cells to the anticancer drugs 5-fluorouracil and doxorubicin through specific modulation of cellular signaling pathways, which is a result of the presence of a hydroxyl group at the C-3 position of the flavonoid core structure in quercetin or rutinose in rutin. Quercetin showed greater cytotoxicity than rutin at the same dose against HCT116 cancer cells and was a better chemosensitizer of doxorubicin than rutin. However, rutin was a better chemosensitizer of 5-fluorouracil than quercetin at the same dose. Doxorubicin was much more cytotoxic than 5-fluorouracil against HCT116 cells, probably due to the absence of protective autophagy.

## 1. Introduction

Colon cancer (CC) is the second and third most common cancer in women and men, respectively, and is among the leading causes of death caused by cancer in the world [1]. Antimetabolite 5-fluorouracil (5-FU) is a popular choice of chemotherapy for a wide range of cancers, particularly CC [2]. Doxorubicin (DOX) is an anthracycline drug, widely used in the treatment of different types of cancers; however, its use in CC is limited due to multi-drug resistance [3]. Both drugs act as RNA- and DNA-damaging agents, inducing oxidative stress and apoptotic death in cancer cells [2,4]. However, antitumor drugs target cells in a non-specific manner, thereby affecting healthy cells as well and causing damage to these. An additional obstacle in antitumor therapy is multidrug resistance acquired by cancer cells [3]. Therefore, efforts are made aimed at reducing the toxicity of antitumor drugs as well as increasing susceptibility to anticancer therapy.

**Figure 1:**
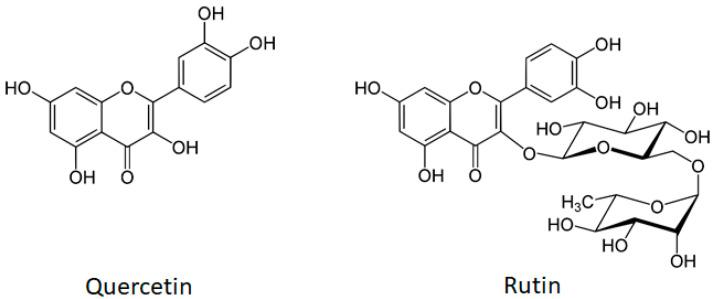
The molecular structures of flavonol quercetin (molecular weight 302.2 g/mol) and flavone rutin (molecular weight 610.5 g/mol). Both compounds have a yellow crystalline appearance, they are sparingly water-soluble and highly soluble in organic solvents). The structure of rutin (quercetin-3-O-rutinoside) consists of flavonol quercetin with the hydroxy group at position C-3 substituted with glucose and rhamnose sugar groups (rutinose).

Natural products are an important source of pharmacologically active compounds [5]. Many of them were identified as potential anticancer drugs and chemosensitizers, showing ability to directly interact with cellular proteins [6,7]. Quercetin and rutin (quercetin-3-O-rutinoside) (Figure 1) are natural compounds that are intensively studied for their therapeutic use in different ailments. Flavonol quercetin and flavone rutin differ in the absence or presence of a hydroxyl group at the C-3 position of the flavonoid backbone, respectively. In rutin, the 3-hydroxyl group is replaced by a disaccharide rutinose. The presence of the 3-hydroxyl group in flavonoids may be a key determinant of their biological activity [8]. We have previously shown different mechanisms of action for the hepatoprotective activity of rutin and quercetin [9].

Both rutin and quercetin possess well-established antioxidant, anti-inflammatory, and anticancer activities [10]. Quercetin has been recently shown to improve the efficacy of 5-FU in CC and melanoma cells [11,12]. Interestingly, previous studies showed that natural compounds, such as resveratrol and curcumin, can overcome multidrug resistance to DOX in CC cells by blocking the activity of P-glycoprotein, an ATP-dependent efflux pump [3,13]. Most recently, quercetin has also been shown as an enhancer of DOX cytotoxicity in P-glycoprotein-overexpressing multiple drug-resistant carcinoma cells [14]. To our knowledge, the chemosensitizing effect of rutin on 5-FU and DOX in CC cells has not been investigated previously.

In the current study, we compared the mechanisms of cytotoxicity of quercetin and its structural analog rutin in CC cells. To investigate their chemosensitization effect, we used two anticancer drugs, 5-FU and DOX. The study objective was to determine the importance of the C-3 position in the anticancer and chemosensitizing activity of these two flavonoids. We determined the cell viability and the expression of proteins which play a key role in oxidative stress, apoptosis, and autophagy.

## 2. Methods

### 2.1. Chemicals and Antibodies

Human colon cancer cell line HCT116 (CCL-247) was obtained from the American Type Culture Collection (Manassas, VA, USA). Quercetin (98%), 5-FU (>99%), and Hoechst 33342 were obtained from Sigma-Aldrich (Steinheim, Germany). Rutin (98.6%) and DOX (>99%) were from Selleckchem (Houston, TX, USA). Radioimmunoprecipitation assay (RIPA) buffer (sc-24948) with protease and phosphatase inhibitors cocktail, and 3-methyladenine (3-MA) were from Santa Cruz Biotechnology (SCB, Santa Cruz, CA, USA). Antibodies to p21 (ab109199), cyclin D1 (ab1663), B-cell lymphoma 2 (Bcl-2) (ab7973), heme oxygenase-1 (HO-1) (ab13243), Akt (ab185633), microtubule-associated protein 1A/1B-light chain 3 beta-I/II (LC3B-I/II) (ab48394), Bcl-2-associated X protein (Bax) (ab32503), and proliferating cell nuclear antigen (PCNA) (ab29) were from Abcam (Cambridge, UK). Antibodies to poly (ADP-ribose) polymerase 1 (PARP1) (#9542), cleaved caspase 3 (Asp175) (#9661), AMP-activated protein kinase (AMPK) (#2535), p-AMPK (#2532), p-Akt (#4060), extracellular signal-regulated kinase 1/2 (ERK1/2), p-ERK1/2, c-Jun N-terminal kinase 1/2/3 (JNK1/2/3), p-JNK1/2, p38, and p-p38 (mitogen-activated protein kinase (MAPK) kit, #9926), forkhead box O3a (FOXO3a) (#2497), p-FOXO3a (Ser294) (#5538), glutathione peroxidase 1 (GPX1) (#3286), GPX4 (#52455), sequestosome 1 (SQSTM1/p62) (#5114), and XTT (2,3-bis(2-methoxy-4-nitro-5-sulfophenyl)-2H-tetrazolium-5-carboxanilide) Cell Viability Assay Kit (#9095) were from Cell Signaling Technologies (CST, Beverly, MA, USA). Glyceraldehyde 3-phosphate dehydrogenase (GAPDH) (HRP-60004) was from Proteintech (Rosemont, IL, USA). Secondary antibodies goat anti-mouse IgG H&L (HRP) (ab97023) and goat anti-rabbit IgG H&L (HRP) (Alexa Fluor 488) (ab150077) were purchased from Abcam. Antibodies against mouse IgG kappa light chain m-IgGκ BP-HRP (sc-516102) were purchased from SCB and anti-rabbit IgG HRP-linked antibody was from CST. All antibodies were validated by the manufacturer. For immunofluorescence, a negative control was used.

### 2.2. Cell Treatments

HCT116 cells were cultured in McCoy’s 5A medium supplemented with 10% fetal bovine serum, 2 mM L-glutamine, streptomycin 10,000 mg/mL, and penicillin 10,000 U/mL (Lonza, Verviers, Belgium) in a CO_2_ incubator (BioSan S-Bt Smart Biotherm, Riga, Latvia). The cells were maintained at 37 °C in a humidified atmosphere of 5% CO_2_. Before starting the treatment, the cells were trypsinized, counted (1 × 10^5^ cells/mL), and seeded. The cells were cultured in McCoy’s 5A medium without antibiotics until approximately 80% confluence and then cultivated in the cell media with tested compounds for 24 h. Rutin, quercetin, and 5-FU were used at concentrations of 200 μM. DOX was used at a 20 μM concentration. The doses were selected based on the preliminary dose-dependent XTT cell viability assay for each compound. To dissolve chemicals, Tween80 and DMSO were used, and full solubility was achieved every time before adding them to the cells. Tween80 and DMSO concentrations in final treatments did not exceed 0.5% (*v*/*v*). Final concentrations of Tween80 and DMSO were added to controls.

### 2.3. Cell Viability Assay

XTT cell viability assay is commonly used for the in vitro determination of cell metabolic activity, which gives an insight into cell viability and proliferation as well as cytotoxicity. The cells were seeded on 96-well plates, and 24 h after the treatments, 50 µL of XTT reagent was added in 200 µL of medium, according to the manufacturer’s instructions. The cells were untreated or treated with rutin, quercetin, DOX, and 5-FU, and with combinations of DOX and 5-FU with rutin and quercetin, respectively. The cells were incubated for 3 h and then absorbance was measured spectrophotometrically at 450 nm (Bio-Tek EL808 Ultra Microplate Reader, BioTek Instruments, Winooski, VT, USA). The reduction of XTT tetrazolium salt to colored formazan detected spectrophotometrically occurs only in metabolically active cells. The metabolic activity of cells that proliferate is higher than that of cells exposed to cytotoxic substances. We performed a preliminary dose-dependent assay with rutin, quercetin, DOX, and 5-FU to determine IC_50_ values. The concentration of each individual compound (rutin, quercetin, DOX, 5-FU) that resulted in 50–80% cell viability was used in further experiments. To study autophagy, the cells were seeded, incubated for 2 h with 5 mM 3-MA, and then treated with rutin, quercetin, DOX, and 5-FU, and with combinations of DOX and 5-FU with rutin and quercetin, respectively, for 24 h, followed by the XTT assay [15,16].

### 2.4. Western Blot

The cell pellets collected after treatments were lysed in 200 μL of ice-cold RIPA buffer containing 50 mM Tris-HCl pH 7.4, 150 mM NaCl, 1% NP-40, 0.5% sodium deoxycholate, 0.1% SDS, with addition of 2 mM phenylmethyl sulphonyl fluoride, 1 mM sodium orthovanadate, and 2 μg/mL of each aprotinin, leupeptin, and pepstatin, with the addition of phosphatase inhibitors on orbital shaker for 2 h, and then centrifuged 15,000 RPM/30 min, after which supernatans were collected [17]. Protein concentration was determined using the Pierce™ Dilution-Free™ Rapid Gold BCA Protein Assay (ThermoFisher Scientific, Waltham, MA, SAD) according to the manufacturer’s instructions. Protein samples were mixed with the sample buffer and then denatured by heating at 95 °C for 5 min. Volume equivalents of 30 or 60 μg of proteins were separated by 8%, 12.5%, or gradient SDS-PAGE and transferred onto the PVDF membrane. The membranes were blocked with non-fat milk in Tris-buffered saline (TBS) with Tween-20 (1:10 *v*/*v*) (TBST) added for 2 h at room temperature and incubated with primary antibodies against p21 (1:1000), cyclin D1 (1:1000), PCNA (1:1000), Bax (1:1000), Bcl-2 (1:1000), caspase-8 (1:1000), cleaved caspase-8 (1:1000), caspase-9/cleaved caspase-9 (1:1000), cleaved caspase-3 (1:250), HO-1 (1:1000), GPX1 (1:1000), GPX4 (1:1000), PARP1 (1:1000), LC3B-I/II (1:500), p62 (1:1000), AMPK (1:1000), p-AMPK (1:1000), Akt (1:1000), p-Akt (1:1000), ERK1/2 (1:1000), p-ERK1/2 (1:1000), JNK1/2/3 (1:1000), p-JNK1/2 (1:500), p38 (1:1000), p-p38 (1:1000), FOXO3a (1:1000), p-FOXO3a (1:1000), and GAPDH (1:10,000) for 2 h at room temperature on an orbital shaker. After incubation, the membranes were washed 3 × 10 min in TBST, following by compatible HRP-linked secondary antibodies for 1 h at room temperature. After washing in TBST 3 × 10 min, the membranes were exposed to a chemiluminescent substrate (SignalFire Elite ECL, Cell Signaling Technologies, Beverly, MA, USA) and scanned by C-DiGit^®^ Blot Scanner (LI-COR Biosciences, Lincoln, NE, USA). A densitometric analysis of bands obtained by Western blotting was performed using ImageJ software(1.54g) [18]. All values were normalized to those of GAPDH for each protein (Supplementary Material File S1).

### 2.5. Immunofluorescence

The cells were grown on round microscopic slides (9161064, Thermo Scientific™, Waltham, MA, USA) in 24-well plates, treated with tested compounds for 24 h, and analyzed for nuclear expression of FOXO3a. After the treatments, the cells were washed, fixed in methanol for 5 min at room temperature, blocked in 5% bovine serum albumin fraction V (A3059, Sigma-Aldrich, Steinheim, Germany) for 2 h and incubated with primary antibodies against FOXO3a (1:250) at 4 °C overnight, washed, and incubated with secondary rabbit green fluorescent protein (GFP) antibodies (1:500), and counterstained with Hoechst 33342. Digital images were acquired by a fluorescence microscope (×1000 magnification) (Olympus IX73, Tokyo, Japan). Measurement of cell fluorescence intensity was performed by ImageJ software [18]. The criteria for fluorescence intensity measurement in color thresholding were based on hue pass as follows: cytosolic (green) ranged from 60° to 120°, and nuclear (blue-green) from 120° to 145°. The color threshold area was divided by the number of cells in the area and it was expressed as a nuclear/cytosolic ratio.

### 2.6. Statistical Analysis

The data presented in the current study (means ± SD) were gathered by performing each test in triplicate and repeating each experiment three times. To establish the effect of different treatment regimens on the cell viability and expression of target proteins, one-way analysis of variance (ANOVA) was used to determine mean square errors within and between groups. Tukey’s post hoc test was used to compare multiple groups: rutin and quercetin compared to control; rutin and quercetin co-treatment with DOX compared to DOX; rutin and quercetin co-treatment with 5-FU compared to 5-FU; DOX and 5-FU treatment compared to control; rutin treatment compared to a similar quercetin treatment. An analysis was carried out with StatSoft STATISTICA 13 (StatSoft Inc., Tulsa, OK, USA). Differences with *p* < 0.05 were considered statistically significant.

## 3. Results

### 3.1. The Cytotoxicity of Rutin, Quercetin, DOX, and 5-FU

**Figure 2:**
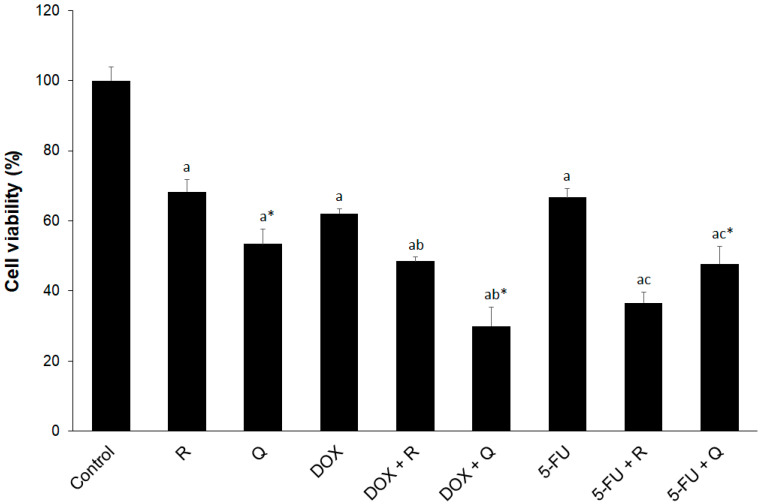
The effect of 200 μM rutin (R), 200 μM quercetin (Q), 20 μM doxorubicin (DOX), and 200 μM 5-fluorouracil (5-FU) and combinations of DOX and 5-FU with rutin and quercetin, respectively, on the viability of HCT116 cells at 24 h. Quercetin showed greater cytotoxicity when compared to rutin. DOX showed greater cytotoxicity than 5-FU, particularly in co-treatment with quercetin, whereas rutin showed greater cytotoxicity than quercetin in co-treatment with 5-FU. The values are mean ± SD, *n* = 3. ^a^ *p* < 0.05 compared to control; ^b^ *p* < 0.05 compared to DOX; ^c^ *p* < 0.05 compared to 5-FU; * *p* < 0.05 Q vs. R treatment.

In order to determine cellular metabolic activity as an indicator of cell viability, proliferation and cytotoxicity, the XTT test was performed on cultured HCT116 cells. In a preliminary dose-dependent test, we determined IC_50_ values for rutin, quercetin, DOX, and 5-FU. IC_50_ for rutin, quercetin, and 5-FU was 354.2, 278.4, and 351.7 µM, respectively, whereas IC_50_ for DOX was 35.8 µM. In order to enable comparative analysis, we chose the same dose (200 µM) for three agents with similar inhibitory potential, 5-FU, rutin, and quercetin, falling into the IC_20–50_ range. We chose 20 µM for DOX since this dose also fitted into the same IC range. The effect of the investigated agents on the viability of HCT116 cells with selected concentrations for further research is presented in Figure 2. Co-treatment with DOX (20 μM) and quercetin (200 μM) and 5-FU (200 μM) and rutin (200 μM) resulted in the highest reduction in cell viability.

### 3.2. The Expression of Antioxidant Enzymes in the Treatments

**Figure 3:**
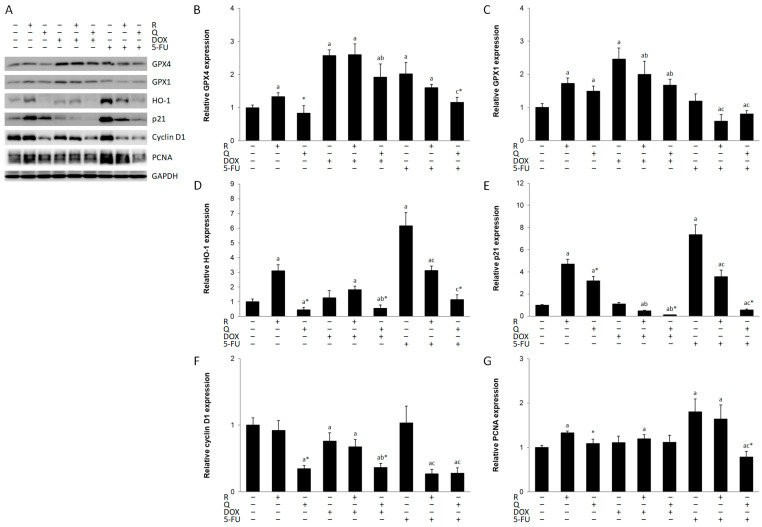
Expression of antioxidant and the cell cycle proteins in HCT116 cell lysates 24 h after treatment with 200 μM rutin (R), 200 μM quercetin (Q), 20 μM doxorubicin (DOX), and 200 μM 5-fluorouracil (5-FU) and combinations of DOX and 5-FU with rutin and quercetin, respectively. Representative blots of glutathione peroxidase (GPX) 1 and 4, heme oxygenase (HO)-1, p21, cyclin D1, and proliferating cell nuclear antigen (PCNA) (**A**). Administration of tested compounds induced changes in the expression of GPX4 (**B**), GPX1 (**C**), HO-1 (**D**), p21 (**E**), cyclin D1 (**F**), and PCNA (**G**). DOX was a strong inducer of both GPX enzymes, whereas 5-FU induced GPX4 and HO-1. R induced all enzymes, while Q induced only GPX1. Protein levels were normalized to GAPDH. The values are mean ± SD, *n* = 3. ^a^ *p* < 0.05 compared to control; ^b^ *p* < 0.05 compared to DOX; ^c^ *p* < 0.05 compared to 5-FU; * *p* < 0.05 Q vs. R treatment.

ROS production and oxidative stress can kill cancer cells and eliminate drug resistance [19]. The results of the current research suggest increased oxidative stress in HCT116 cells by different treatments, as shown by induction of antioxidant enzymes (Figure 3A). Rutin induced all enzymes, while quercetin induced only GPX1. DOX was a strong inducer of both GPX enzymes (Figure 3B,C) but not HO-1, whereas 5-FU induced GPX4 and HO-1 (Figure 3D). Neither rutin nor quercetin treatment contributed to the induction of these antioxidant enzymes by DOX or 5-FU.

### 3.3. The Effect of the Treatments on Cell Cycle Proteins

The cellular response to damaged DNA is arrest of cell cycle progression and promotion of DNA repair [20]. Our results have shown that the cytotoxicity of all studied compounds was accompanied by reduced expression of cyclin D1 (Figure 3F), with the exception of rutin- and 5-FU-treated cells. However, p21 was overexpressed in these two treatments (Figure 3E). Rutin did not decrease cyclin D1 expression in DOX treatment but markedly reduced it in 5-FU treatment. However, quercetin readily suppressed cyclin D1 expression in both DOX and 5-FU treatments. Both rutin and quercetin markedly increased p21 expression, but both compounds suppressed p21 expression in DOX and 5-FU treatments. The expression of PCNA was also higher in rutin and 5-FU treatments and in their co-treatment (Figure 3G).

### 3.4. The Activation of Apoptotic Cell Death and Autophagy

**Figure 4:**
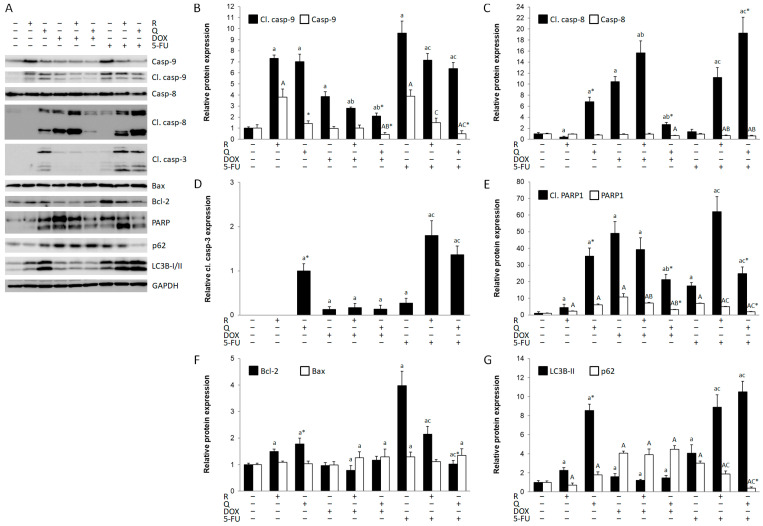
Expression of apoptotic and autophagic proteins in HCT116 cell lysates 24 h after treatment with 200 μM rutin (R), 200 μM quercetin (Q), 20 μM doxorubicin (DOX), and 200 μM 5-fluorouracil (5-FU) and combinations of DOX and 5-FU with rutin and quercetin, respectively. Representative blots of caspase-9, caspase-8, caspase-3, Bax, Bcl-2, poly (ADP-ribose) polymerase (PARP)1, and microtubule-associated protein 1A/1B-light chain 3 beta (LC3B)-II expression (**A**). Administration of tested compounds induced changes in the expression of caspase-9 (**B**), caspase-8 (**C**), caspase-3 (**D**), PARP1 (**E**), Bax and Bcl-2 (**F**), and LC3B-II and p62 (**G**). DOX and Q induced both extrinsic and intrinsic, while 5-FU and R induced only intrinsic apoptotic cell death. Autophagy was increased in R and Q treatments and particularly in their co-treatment with 5-FU. Protein levels were normalized to GAPDH. The values are mean ± SD, *n* = 3. ^a, A^ *p* < 0.05 compared to control; ^b, B^ *p* < 0.05 compared to DOX; ^c, C^ *p* < 0.05 compared to 5-FU; * *p* < 0.05 Q vs. R treatment.

Cancer cell cytotoxicity is often caused by apoptotic cell death. Activated caspase-9 was induced by rutin and quercetin, as well as DOX and 5-FU; however, both rutin and quercetin decreased the expression of cleaved caspase-9 compared to DOX and 5-FU treatments (Figure 4A,B). In contrast, DOX, but not 5-FU, activated caspase-8 (Figure 4C). Rutin additionally increased the activation of caspase-8 in DOX treatment. Quercetin, but not rutin, activated caspase-8, acting synergistically with 5-FU. Caspase-3 was activated in all treatments, except by rutin. Quercetin and co-treatment with 5-FU and both rutin and quercetin were the strongest activators of caspase-3 (Figure 4D). Nevertheless, PARP1 was cleaved in all treatments (Figure 4E). Quercetin was a stronger inducer of PARP1 cleavage than rutin, but rutin in co-treatment with DOX and 5-FU resulted in higher levels of cleaved PARP1 than quercetin. DOX was a strong inducer of PARP1 cleavage than rutin, which does not follow activated caspase-3 expression, suggesting treatment-dependent induction of apoptosis by caspase-3-dependent and -independent mechanisms. Bax expression remained constant during the experiment (Figure 4F), while Bcl-2 expression was increased by the rutin and quercetin treatments and particularly by the 5-FU treatment (Figure 4F). Autophagy is also frequently upregulated during anti-cancer drug treatment. The expression of LC3B-II, a key protein in autophagosome formation, was more increased by quercetin than by rutin (Figure 4G), as well as by 5-FU alone, and particularly by 5-FU in combined treatments with rutin and quercetin. The expression of autophagy substrate p62 was increased in all treatments except with rutin and co-treatment with 5-FU and quercetin (Figure 4G). The highest expression of p62 was in all DOX treatments, while LC3B-II expression was close to normal in these treatments.

### 3.5. The Modulation of Key Signaling Pathways

**Figure 5:**
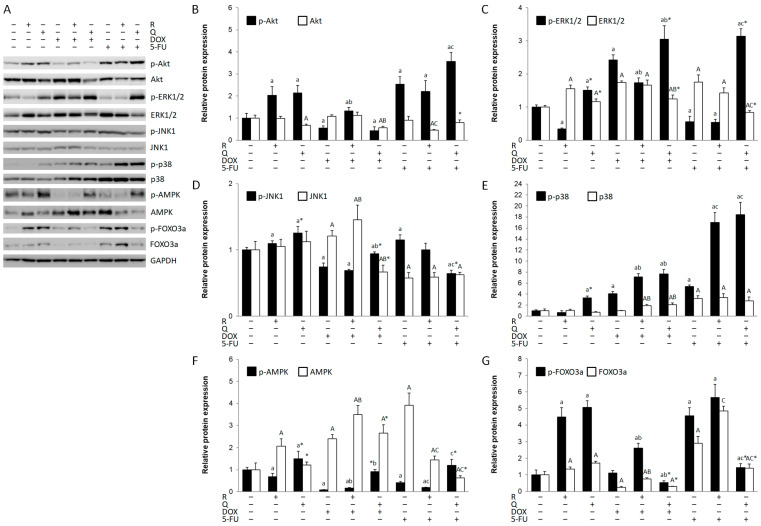
Expression of key signaling molecules in HCT116 cell lysates 24 h after treatment with 200 μM rutin (R), 200 μM quercetin (Q), 20 μM doxorubicin (DOX), and 200 μM 5-fluorouracil (5-FU) and combinations of DOX and 5-FU with rutin and quercetin, respectively. Representative blots of Akt, MAPKs, AMPK, and FOXO3a expression (**A**). Treatment with tested compounds induced changes in Akt (**B**), ERK1/2 (**C**), JNK1/2 (**D**), p38 MAPK (**E**), AMPK (**F**), and FOXO3a activation (**G**). The most pronounced changes were in p-p38 expression, which was increased in all treatments except with R, particularly in 5-FU co-treatment with Q and R. Activation of AMPK was mainly reduced in different treatments, particularly in DOX and 5-FU treatments and their co-treatment with R. Protein levels were normalized to GAPDH. The values are mean ± SD, *n* = 3. ^a, A^ *p* < 0.05 compared to control; ^b, B^ *p* < 0.05 compared to DOX; ^c, C^ *p* < 0.05 compared to 5-FU; * *p* < 0.05 Q vs. R treatment.

Apoptosis and autophagy are tightly regulated by upstream signaling pathways. Thus, we sought to determine the role of the PI3K/Akt, MAPK, AMPK, and FOXO3a pathways in HCT116 cytotoxicity (Figure 5A). Rutin activated Akt (Figure 5A,B), JNK1/2 (Figure 5D), and FOXO3a (Figure 5G) and additionally increased p38 (Figure 5E) and FOXO3a in co-treatment with DOX and 5-FU, as well as ERK1/2 (Figure 5C) in co-treatment with DOX. The expression of AMPK (Figure 5F) was downregulated in all rutin treatments. Quercetin induced all key signaling pathways (Figure 5D) and additionally induced Akt, ERK1/2, and p38 in combined treatment with 5-FU. Quercetin activated AMPK when compared to the control and rutin-treated cells, as well as DOX and 5-FU and their combined treatment with rutin. DOX activated ERK1/2 and p38 MAPK, while other pathways were downregulated, except FOXO3a. 5-FU strongly activated Akt (Figure 5B), p38 MAPK (Figure 5E), and FOXO3a (Figure 5G), while ERK1/2 (Figure 5C) was suppressed. DOX and 5-FU markedly suppressed p-AMPK expression compared to the control.

### 3.6. Autophagy Played a Dominantly Protective Role in the Treatments

**Figure 6:**
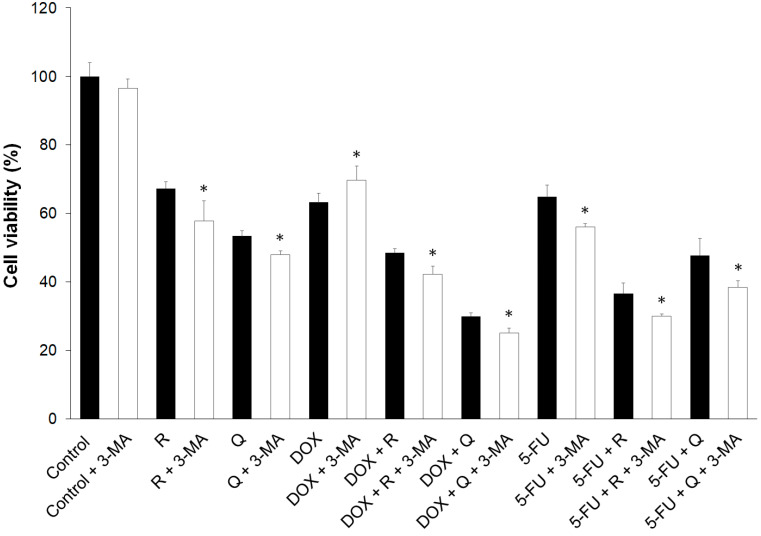
The effect of 3-methyladenine (3-MA) on the viability of HCT116 cells treated for 24 h with 200 μM rutin (R), 200 μM quercetin (Q), 20 μM doxorubicin (DOX), and 200 μM 5-fluorouracil (5-FU) and combinations of DOX and 5-FU with Q and R, respectively. The addition of 3-MA to untreated control and individual treatments and co-treatments significantly reduced cancer cell viability, except for DOX treatment. The values are mean ± SD, *n* = 3. * *p* < 0.05 vs. 3-MA treatment.

Autophagy can induce cytotoxicity as an alternative form of cell death; however, autophagy can also rescue cells from death by removal of damaged cell components and organelles. The results of the current study showed a decrease in the viability of the cells treated with autophagy inhibitor 3-MA, except in control cells, and an increase in cell viability with DOX treatment (Figure 6). 3-MA acts as an inhibitor of autophagic flux by blocking autophagosome formation via the inhibition of type III PI3Ks [16]. The decrease in cell viability following 3-MA treatment suggests that autophagy was required for cell survival, and in the case of DOX, the increase in cell viability suggests cytotoxic autophagy.

### 3.7. HCT116 Treatments Induced FOXO3a Nuclear Accumulation

**Figure 7:**
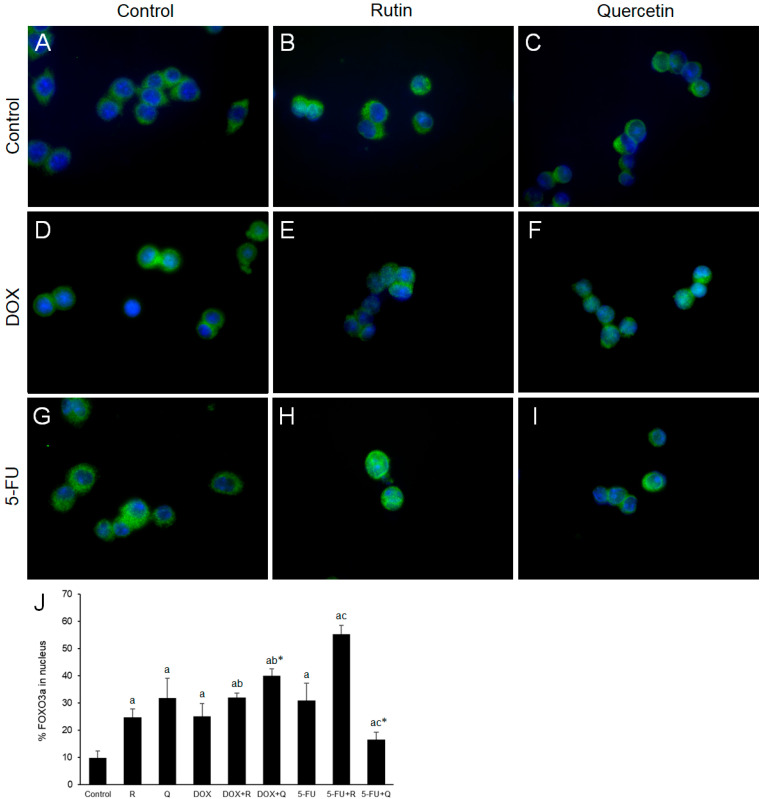
Representative images of GFP immunostaining (green) of FOXO3a nuclear expression 24 h after treatments. FOXO3a was mainly localized in the cytoplasm of control cells (**A**) with an increase in nuclear FOXO3a in cells treated with 200 μM rutin (R) and 200 μM quercetin (Q) was observed ((**B**) and (**C**), respectively). DOX (**D**) 20 μM and its co-treatment with R (**E**) and Q (**F**) showed significant nuclear localization of FOXO3a compared to control. Treatment with 5-FU 200 μM also increased nuclear immunopositivity of FOXO3a (**G**), which was markedly enhanced by treatment with R (**H**), but not Q (**I**). Nuclei are stained with Hoechst (blue) in all images. Quantification of the nuclear content of FOXO3a (**J**). Original magnification ×1000. The values are mean ± SD, *n* = 3. ^a^ *p* < 0.05 compared to control; ^b^ *p* < 0.05 compared to DOX; ^c^ *p* < 0.05 compared to 5-FU; * *p* < 0.05 Q vs. R treatment.

Transcription factor FoxO3a functions as a tumor suppressor by regulating expression of genes involved in oxidative stress, cell cycle arrest, apoptosis, and autophagy [21]. Immunofluorescence microscopy and measurement of fluorescence intensity (Figure 7J) showed the presence of nuclear FOXO3a in untreated cells (Figure 7A). Rutin (Figure 7B), quercetin (Figure 7C), DOX (Figure 7D), 5-FU (Figure 7G) and co-treatment with DOX and either rutin or quercetin (Figure 7E,F) increased the nuclear content of FOXO3a. Co-treatment with 5-FU and rutin induced the highest increase (Figure 7H) in FOXO3a nuclear expression compared to control, while co-treatment with 5-FU and quercetin (Figure 7I) resulted in a minimal increase.

## 4. Discussion

The results of the current study showed that both rutin and quercetin exhibit cytotoxic effects and chemosensitize HCT116 CC cells to 5-FU and DOX. This is of particular importance for DOX, which was cytotoxic at a 10-fold lower dose than 5-FU, and for which various attempts are being made to overcome its resistance in the treatment of CC [22,23,24,25]. The chemosensitization of cancer cells by rutin and quercetin appears to be mediated through their ability to modulate multiple cell-signaling molecules, such as those involved in cell survival and proliferation. Table 1 summarizes the effect of each treatment and co-treatment on key cellular processes.

The survival of cancer cells depends on multiple factors, including cellular redox status. Oxidative stress plays an important role in the initiation and development of CC [26]. The antioxidant selenoenzymes GPX1 and GPX4 play a fundamental role in metabolizing intracellular peroxides such as hydrogen peroxide and lipid hydroperoxides [27,28]. GPX4 was identified as a central negative regulator of ferroptosis, a unique iron and lipid peroxidation-dependent type of cell death [29]. The increase in GPX4 expression in the current study suggests that rutin, DOX, and 5-FU protected the cells from ferroptosis. Another antioxidant enzyme, HO-1, plays a dual role in cancer, preventing the cancer cells from apoptosis and autophagy, but also inducing ferroptosis, which is generally accompanied by GPX4 inhibition [30]. However, since both apoptosis and autophagy can be triggered by oxidative stress [31], we suggest that the increase in HO-1 was insufficient to suppress both processes. Moreover, since autophagy played a protective role, except in DOX treatment, the increase in autophagy can be attributed to an attempt to remove existing oxidative damage [32]. GPX1 has been shown to induce a protective autophagy [28]. The highest expression of GPX1 in the current study was found in treatment with DOX, supporting previous findings.

Cyclin D1, the regulator of the cell cycle progression, is commonly overexpressed in CC cells, acting mainly as an oncogenic driver [33]. In the current study, cyclin D1 remained unchanged in rutin and 5-FU-treated cells and was decreased in other treatments compared to controls. However, the expression of the cell cycle inhibitor p21 was highly induced in rutin- and 5-FU-treated cells. Interestingly, it has been suggested that p21 induction may significantly contribute to the response of CC cells to DOX treatment [34]. However, in the current study, the expression of p21 was steady in DOX treatment and even reduced in its co-treatment with rutin and quercetin, although the viability of cancer cells was markedly reduced. This suggests that induction of p21 is not obligatory for cytotoxicity induced by DOX. p21 can also be associated with PCNA, a torus-shaped homotrimeric DNA-interacting protein known for multiple functions, including cell-cycle regulation, DNA replication, and DNA repair [35]. The interplay between p21 and PCNA is complex, but the most accepted function of PCNA is the control of DNA replication by interaction with p21 [35]. Since rutin, 5-FU, and 5-FU + rutin induced both p21 and PCNA, the cytotoxic and 5-FU-sensitizing effect of rutin could occur through the cell cycle arrest and impairment of replicative DNA synthesis.

If cell cycle arrest is not sufficient to repair damaged DNA, the cell undergoes apoptotic cell death [36]. CC is characterized by the partial suppression of apoptosis, although in advanced stages of CC, the spontaneous induction of apoptosis may contribute to tumor growth [37]. Two distinct apoptotic pathways, intrinsic and extrinsic, differ in the activation of specific caspases and specific cleavage targets [38]. Extrinsic apoptotic pathways can be induced by membrane receptors, such as TRAIL and Fas, which lead to activation of caspase-8, whereas intrinsic cell death is mediated by mitochondrial cytochrome c release and activation of caspase-9 [38]. In the current study, rutin and DOX strongly induced intrinsic, and 5-FU induced extrinsic apoptotic cell death. Caspase-3 in a common executioner caspase and its proteolytic activation results in the cleavage of various target proteins, including PARP1 [39]. PARP1 is involved in the repair of DNA damage in response to a variety of cellular stresses. During apoptosis, PARP1 is cleaved and inactivated, preventing its protective action. In the current study, rutin and 5-FU induced the lowest response to apoptosis (Bcl-2, cleaved caspase-3, PARP1) compared to other treatments. However, apoptosis, with a concomitant reduction in the cell viability, markedly increased by 5-FU and rutin co-treatment, suggesting a specific chemosensitization of the cells to 5-FU by rutin. Programmed cell death is under the control of the PI3K/Akt, MAPK, and AMPK pathways [40,41]. Chemosensitization of DOX by quercetin was associated with induction of ERK 1/2 and p38 MAPK, while chemosensitization of 5-FU with rutin was accompanied by induction of Akt and p38 MAPK.

Autophagy is a self-digestion process through which cells decompose and reuse unnecessary or dysfunctional cellular components [42]. The PI3K/Akt, MAPK, and AMPK pathways also act as crucial regulators of autophagy [43]. The expression of LC3B-II, a key structural protein of autophagosome membranes [44], which was most intense in treatment with 5-FU and its co-treatment with rutin and quercetin, suggested ongoing autophagy in all except for DOX treatments. Interestingly, the results obtained with the autophagy inhibitor 3-MA indicated the cytotoxic role of autophagy in DOX treatment. Sequestosome 1 (p62/SQSTM1) is a classical selective autophagy receptor and p62 levels are used as a marker for autophagic flux, along with LC3B [45]. In general, total cellular expression levels of p62 inversely correlate with autophagic activity [46]. The accumulation of p62 in all DOX treatments, together with low LC3B-II levels, indicated defective autophagy. Taken together, we suggest that autophagy has played a negligible role in cell death by DOX treatment and its co-treatments.

FOXO3a plays a dual role in cancer survival, regulating the transcription of proteins involved in the cell cycle, apoptosis, cellular metabolism, and autophagy. It is frequently considered a tumor suppressor [12]. However, in CC cells, although FOXO3a mediated the cytotoxic effect of cisplatin, it protected cells against DOX cytotoxicity [47,48]. FOXO3a is phosphorylated by upstream regulators (Akt, ERK1/2, JNK1), inducing its inactivation and translocation from the nucleus to the cytoplasm. In contrast, phosphorylation of FOXO by AMPK and p38 promotes its entry into the nucleus, which stimulates the transcriptional activity of FOXO [12]. FOXO regulates different targets, including up-regulation of p21 and down-regulation of cyclin D1 [49]. Phosphorylation at Ser294 could be mediated by ERK1/2, p38, or JNK1 [50,51]. In the current study, it seems that p38 was responsible for FOXO3a (Ser294) phosphorylation, similar to our previous findings [15]. The cytotoxicity of all tested compounds was accompanied by increased FOXO3a nuclear entry. The highest FOXO3a expression was observed in DOX and quercetin, as well as 5-FU and rutin co-treatments, coinciding with the lowest cell viability, suggesting that FOXO3a was involved in the cancer cell cytotoxicity by these treatments.

### Immediate Future Directions

Keeping in mind that cancer cell lines do not exhibit the same complexities as in vivo systems, further extensive studies with animal models are required.

## 5. Conclusions

The results of the current study showed that both rutin and quercetin possess specific effects on the cytotoxicity and chemosensitization of CC cells through specific modulation of cell signaling pathways, which is the result of the presence of a hydroxyl group at the C-3 position of the core flavonoid structure in quercetin or rutinose in rutin. Quercetin showed greater cytotoxicity than rutin at the same dose against HCT116 cancer cells and was a better chemosensitizer of DOX than rutin. However, rutin was a better chemosensitizer of 5-FU than quercetin at the same dose. DOX was much more cytotoxic than 5-FU against HCT116 cells, probably due to the absence of protective autophagy. This suggests the necessity of reassessing DOX as a part of a combination therapy for CC, including quercetin as a strong chemosensitizer of CC cells. The results of this study highlight the translational potential of flavonoids in cancer therapeutics and the need for further clinical studies. The combination of compounds with different mechanisms of action could contribute to the efficacy of cancer therapeutics through chemosensitization, reduced dosage, and decreased susceptibility to drug resistance.

## Figures and Tables

**Table 1:** The effect of different treatments on apoptosis, autophagy, and signaling pathways in HCT116 CC cells. R, rutin; Q, quercetin; DOX, doxorubicin; 5-FU, 5-fluorouracil. 0, no effect; +/−, low effect; ++/−−, moderate effect; +++/−−−, high effect, compared to control.

Cellular Event	Treatment								
	Control	R	Q	DOX	DOX + R	DOX + Q	5-FU	5-FU + R	5-FU + Q
Apoptosis	−	+	+++	+++	+++	++	++	+++	++
Autophagy	−	+	+++	+	+	+	++	+++	+++
Akt activation	−	++	++	−	+	−	++	++	+++
ERK activation	−	−−	+	++	+	+++	−−	−−	+++
JNK activation	−	+	+	−−	−−	−	+	0	++
P38 activation	−	0	+	+	++	++	+	+++	+++
AMPK activation	−	−	+	−−−	−−−	0	−−−	−−	0
Nuclear FOXO3a	−	++	++	++	++	+++	++	+++	+

## Data Availability

The raw data supporting the conclusions of this article will be made available by the authors on request.

## Supplementary Materials

The following supporting information can be downloaded at: https://www.mdpi.com/article/10.3390/biology14050527/s1, File S1: The full uncropped blots images.

## Abbreviations

3-MA: 3-methyladenine; 5-FU, 5-fluorouracil; AMPK, AMP-activated protein kinase; Bax, Bcl-2-associated X protein; Bcl-2; B cell lymphoma 2; DOX, doxorubicin; ERK, extracellular signal-regulated kinase; FOXO, forkhead box O; HO, heme oxygenase; GPX, glutathione peroxidase; GAPDH, glyceraldehyde 3-phosphate dehydrogenase HRP, horseradish peroxidase; JNK, c-Jun N-terminal kinase; LC3B, microtubule-associated protein1A/1B-light chain 3 beta; MAPK, mitogen-activated protein kinase; PARP, poly (ADP-ribose) polymerase; PCNA, proliferating cell nuclear antigen; PI3K, phosphoinositide 3-kinase; PI3K, phosphoinositide 3-kinase; PVDF, polyvinylidenefluoride; RIPA, radioimmunoprecipitation assay; ROS, reactive oxygen species; RPM, rounds per minute; SDS-PAGE, sodium dodecyl sulfate-polyacrylamide gel electrophoresis; XTT, 2,3-bis(2-methoxy-4-nitro-5-sulfophenyl)-2H-tetrazolium-5-carboxanilide.
