# Supplementary material for: Differential Effects of Rutin and Its Aglycone Quercetin on Cytotoxicity and Chemosensitization of HCT 116 Colon Cancer Cells to Anticancer Drugs 5-Fluorouracil and Doxorubicin
Source: Biology (Basel). 2025 May 9;14(5):527. doi: 10.3390/biology14050527 (PMC12109564; doi:10.3390/biology14050527)

Supplementary Materials

Journal: Biology

Title: Differential Effects of Rutin and Its Aglycone Quercetin on Cytotoxicity and Chemosensitization of HCT 116 Colon Cancer Cells to Anticancer Drugs 5-Fluorouracil and Doxorubicin

Iva Suman, Alberta Jezidžić, Dorotea Dobrić and Robert Domitrović

Prof. Robert Domitrović, Ph. D., DDepartment of Medical Chemistry, Biochemistry and Clinical Chemistry, Faculty of Medicine, University of Rijeka, Braće Branchetta 20, 51000 Rijeka, Croatia. E-mail: robert.domitrovic@uniri.hr

The full uncropped blots images.

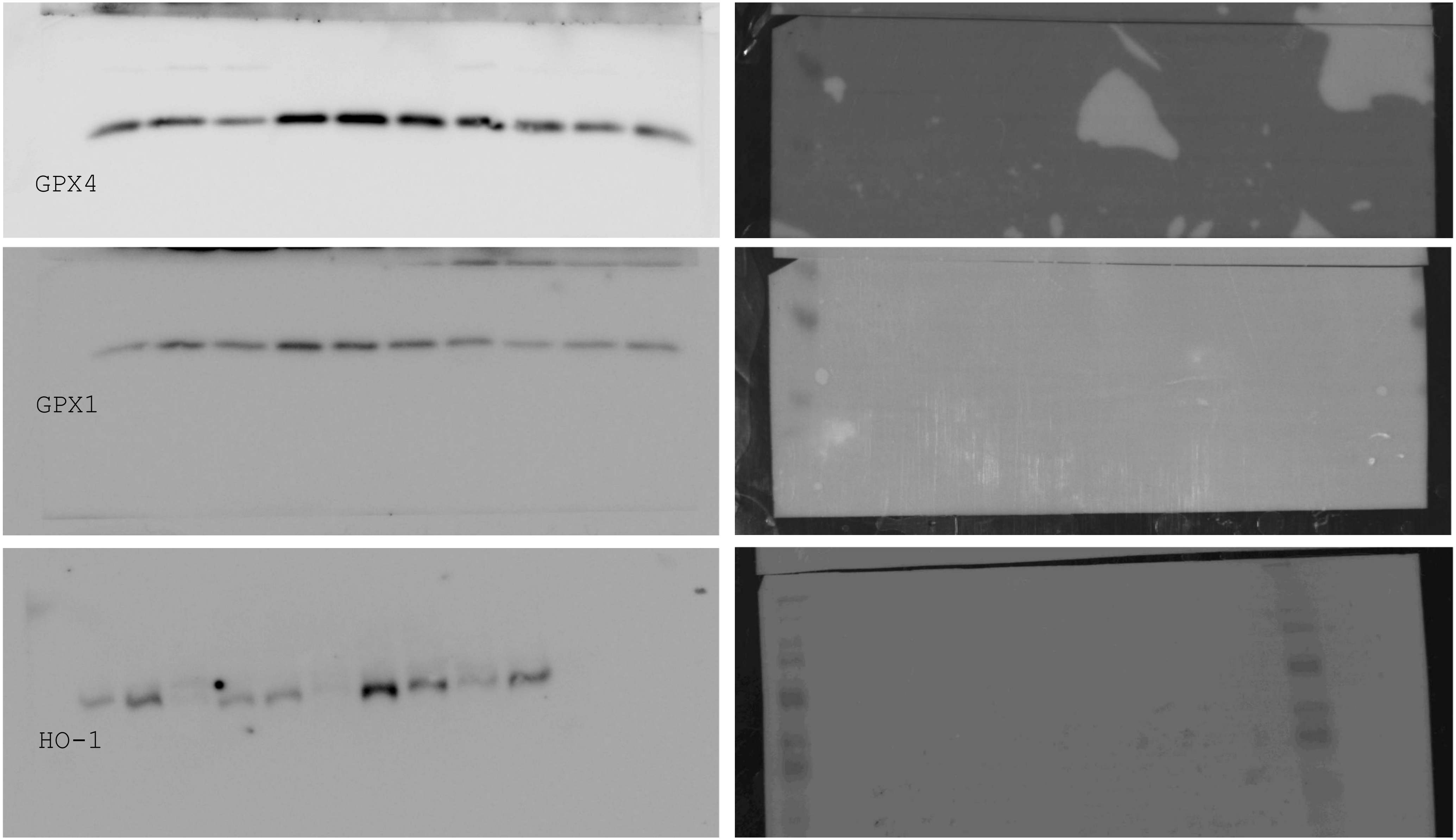
GPX4
GPX1
HO-1

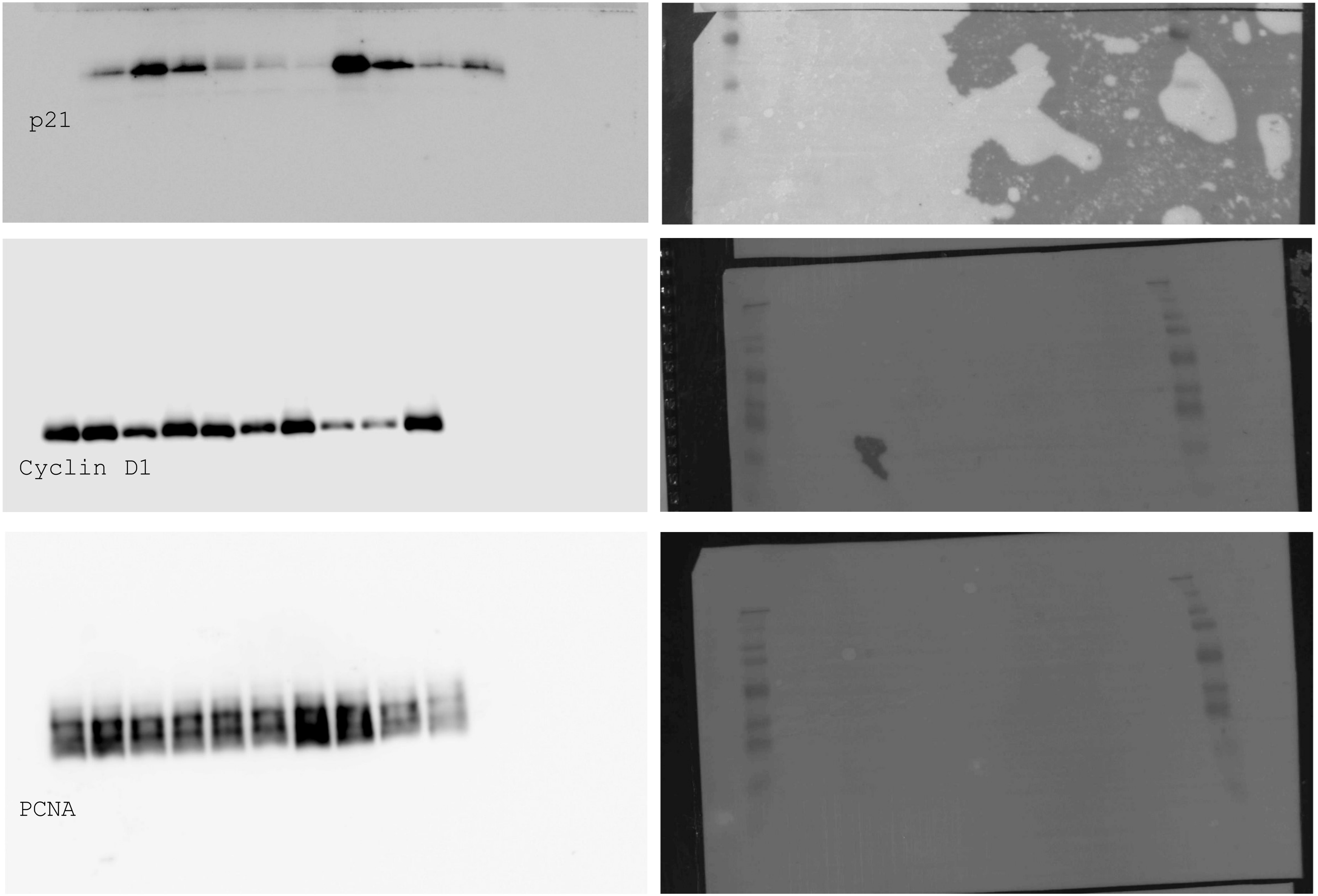
p21
Cyclin D1
PCNA

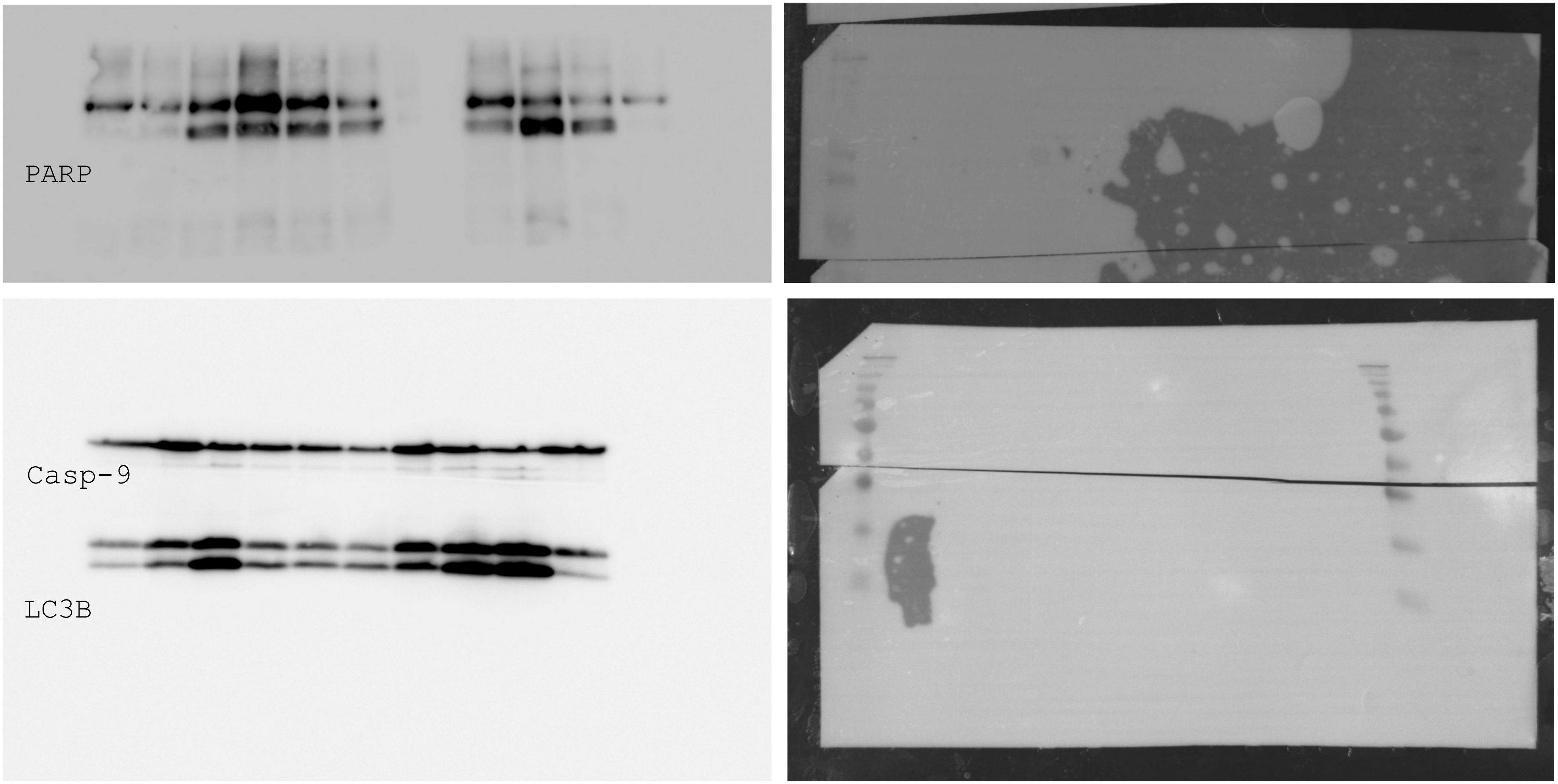
PARP
Casp-9
LC3B

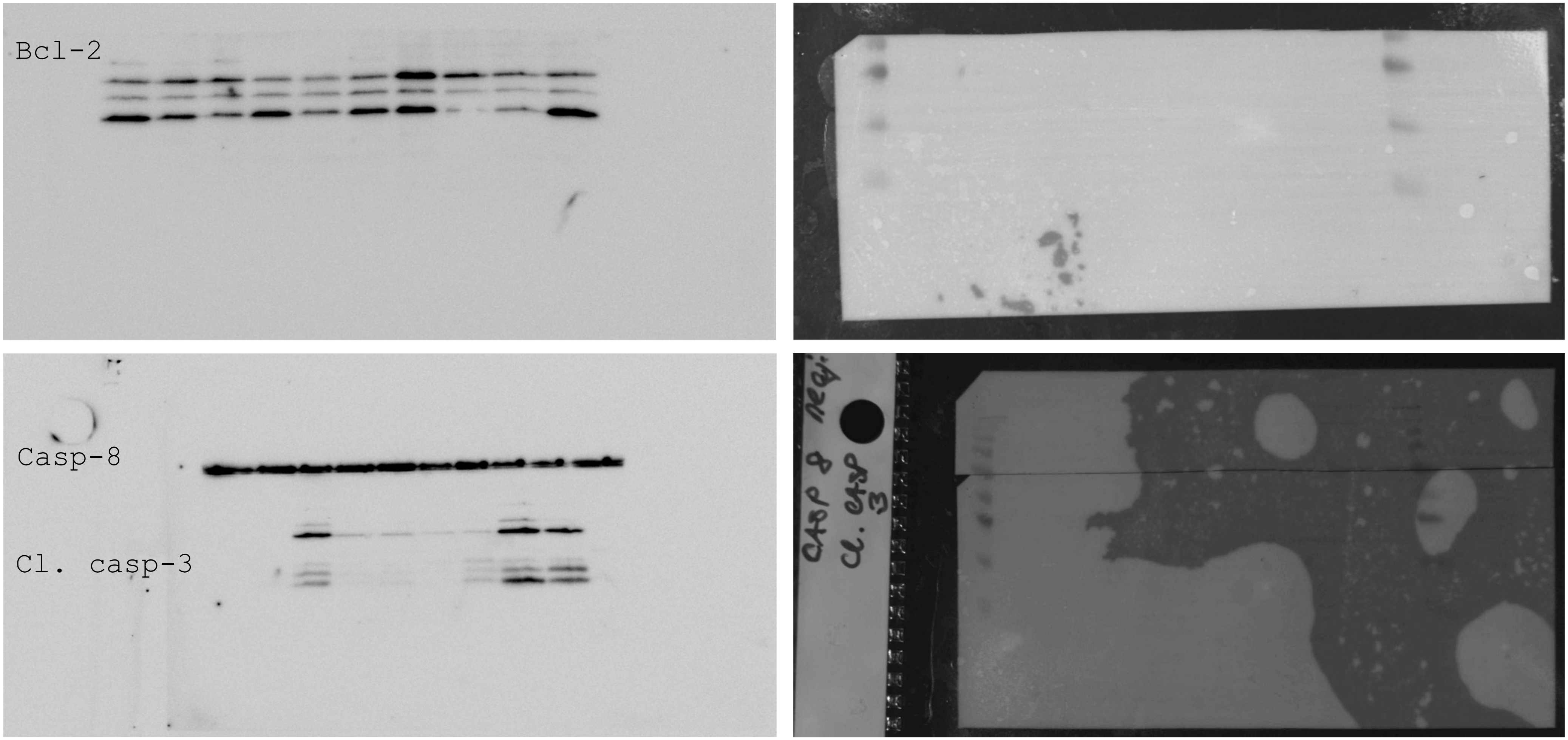
Bcl-2
Casp-8
Cl. casp-3

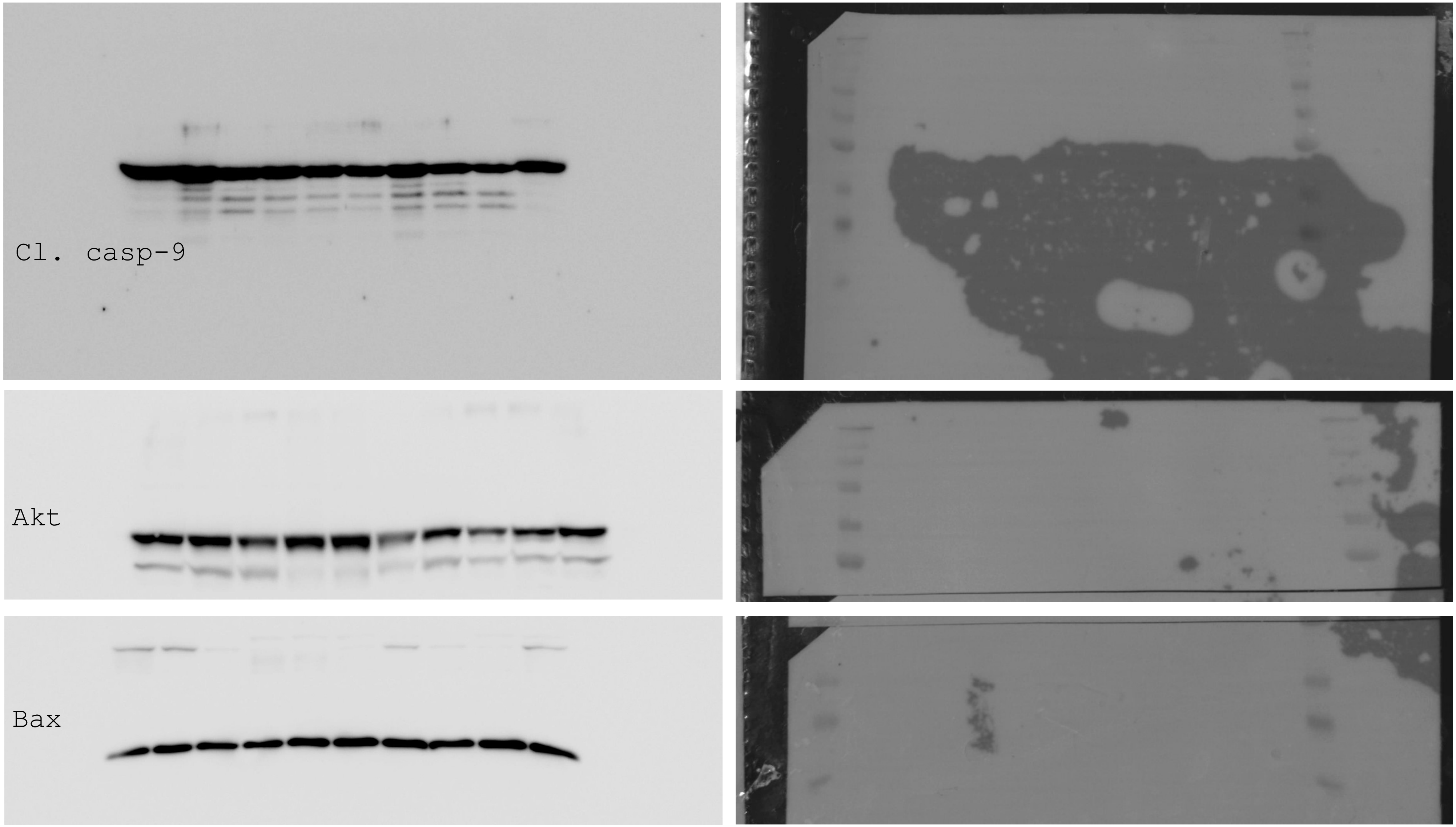
Cl. casp-9
Akt
Bax

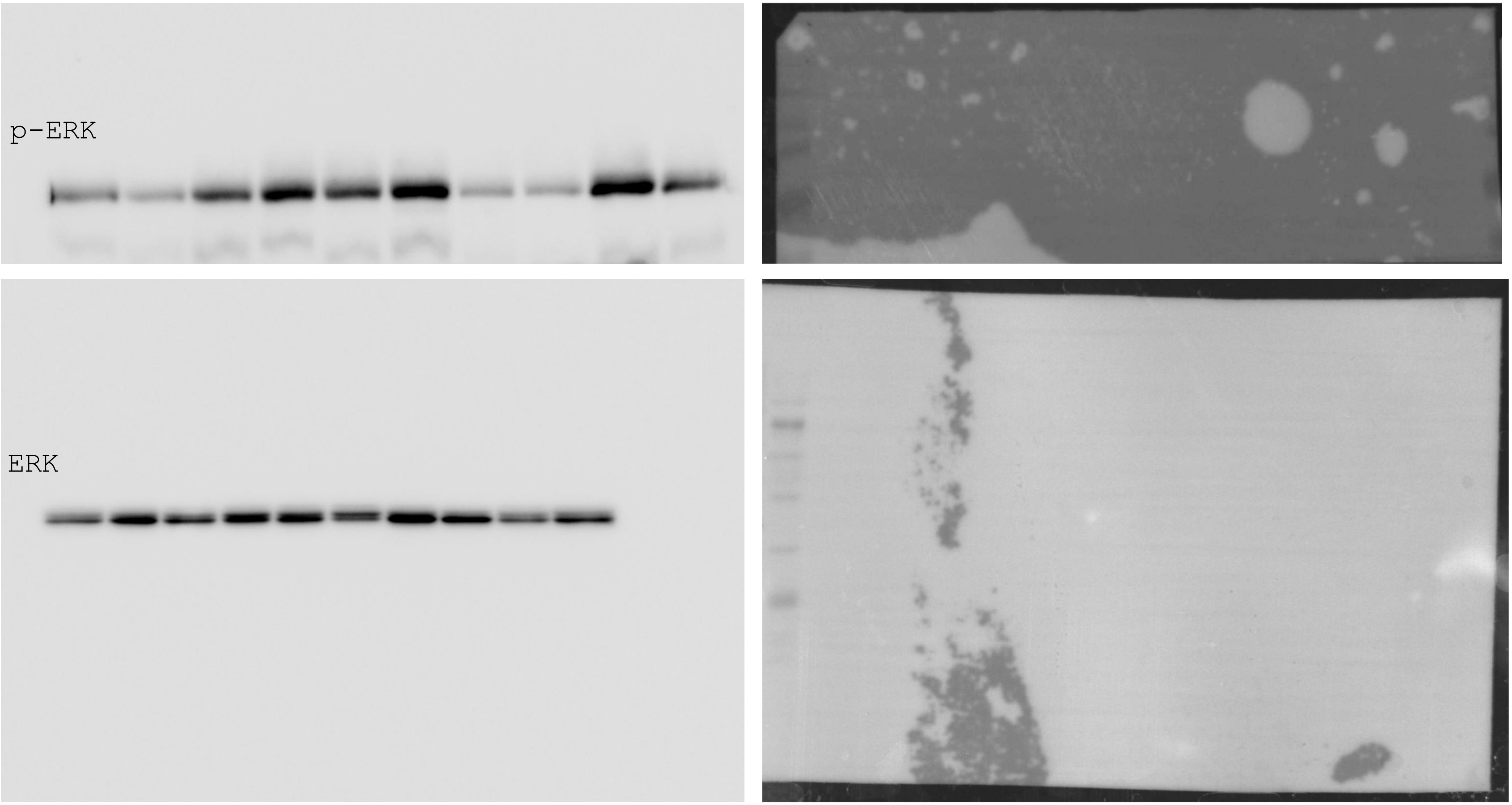
p-ERK
ERK

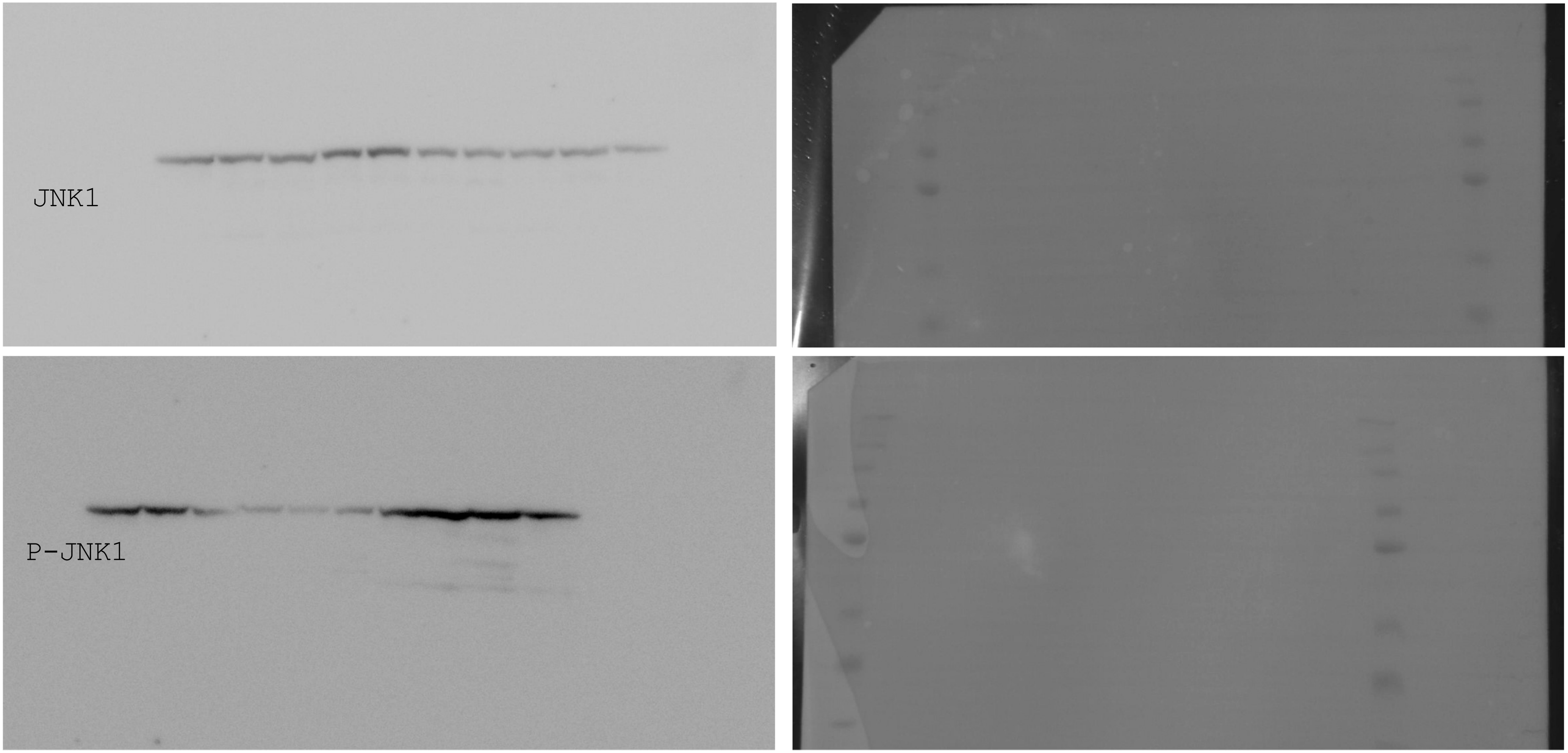
JNK1
P-JNK1

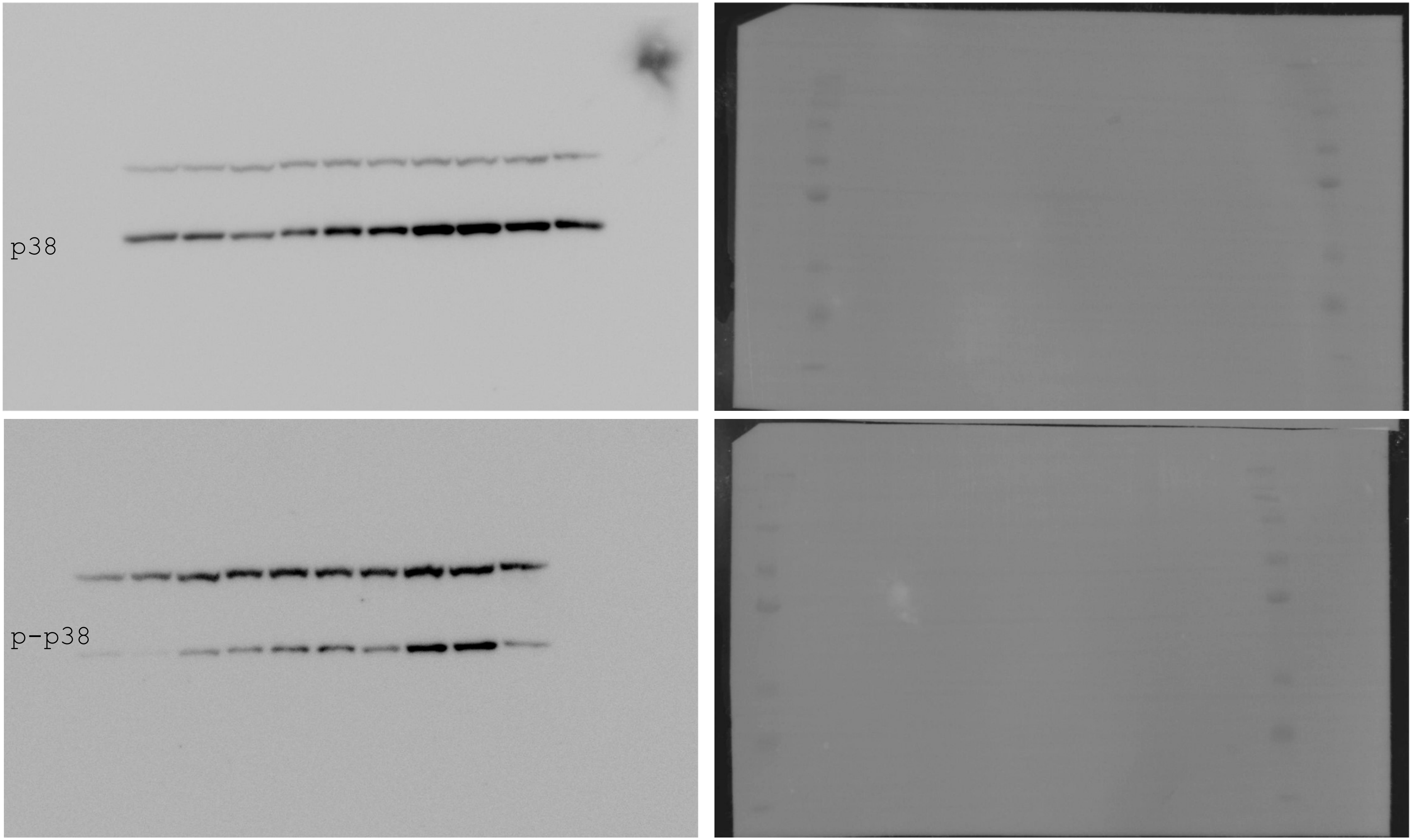
p38
p-p38

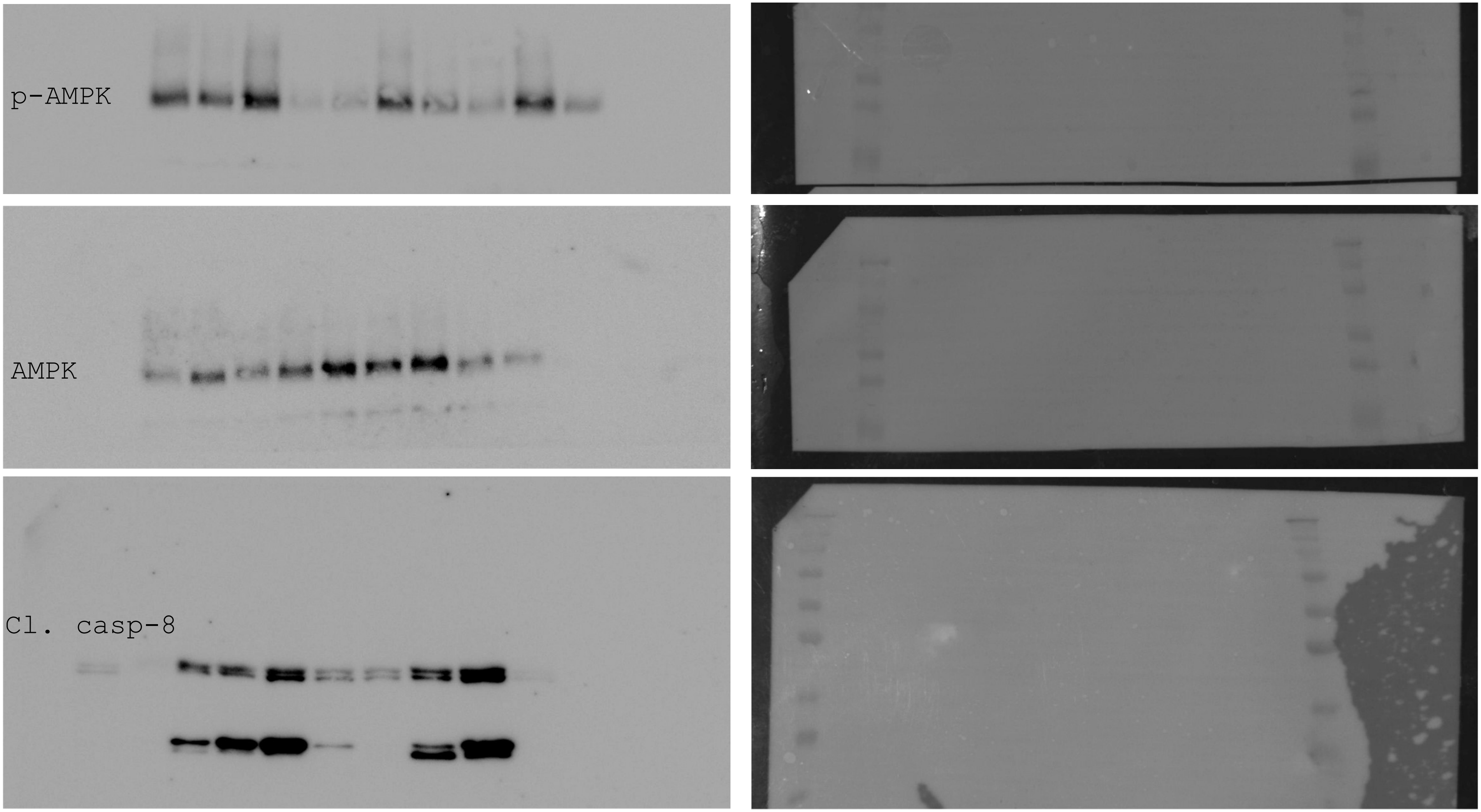
p-AMPK
AMPK
Cl. casp-8

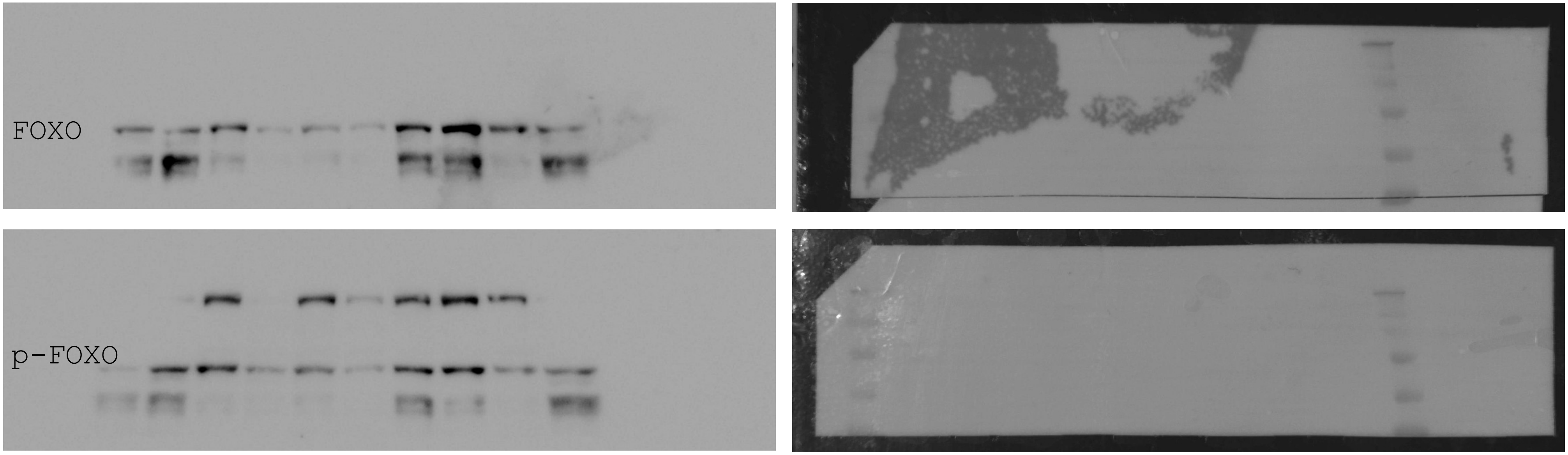
FOXO
p-FOXO

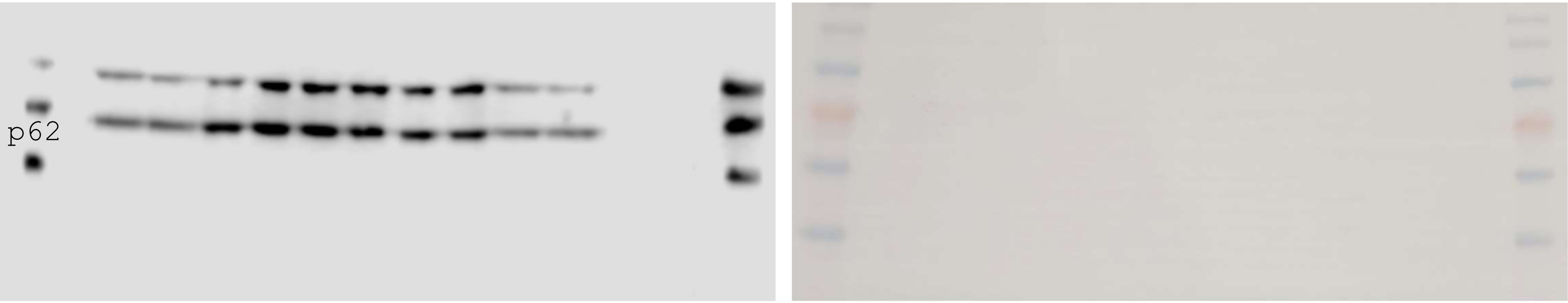
p62

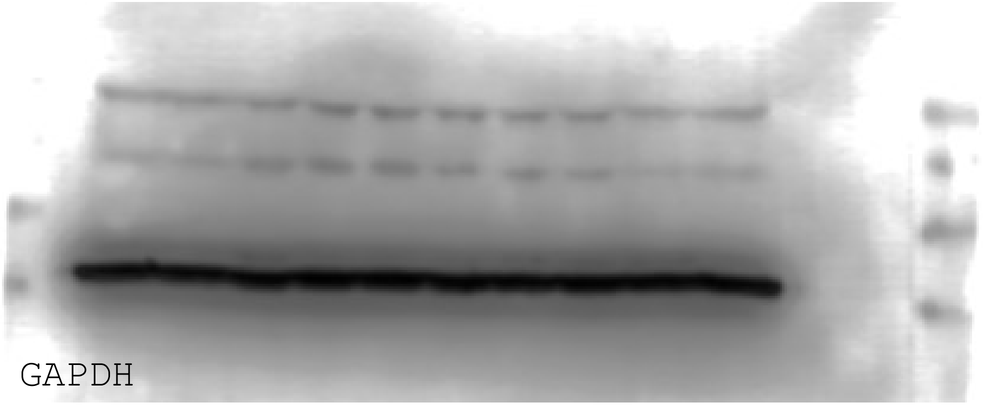
GAPDH
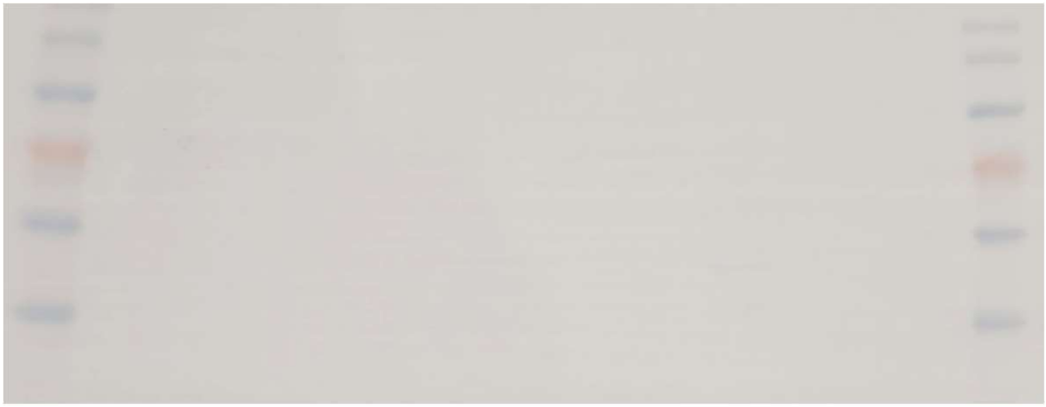
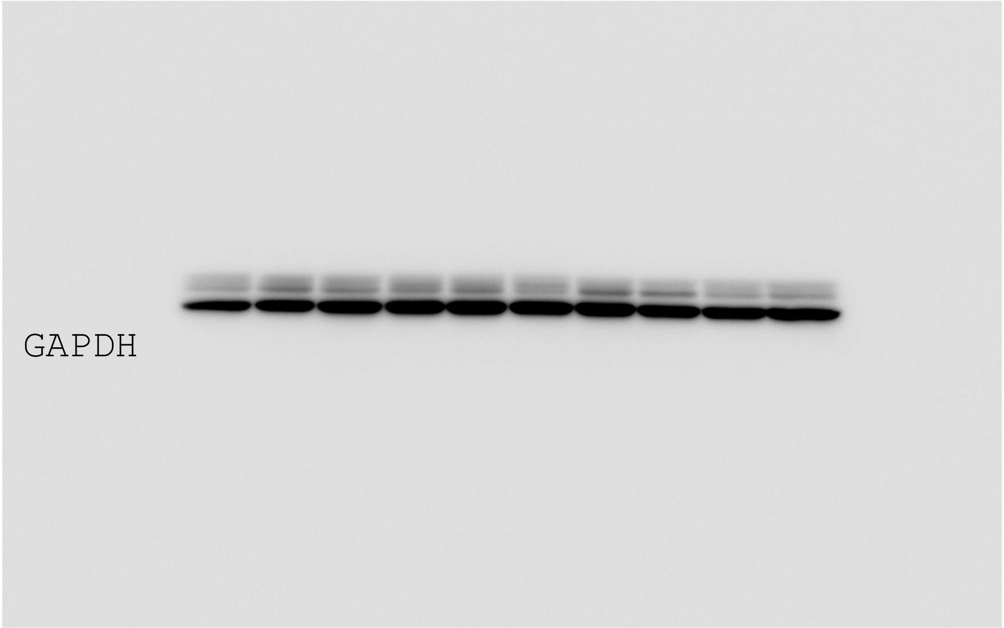
GAPDH
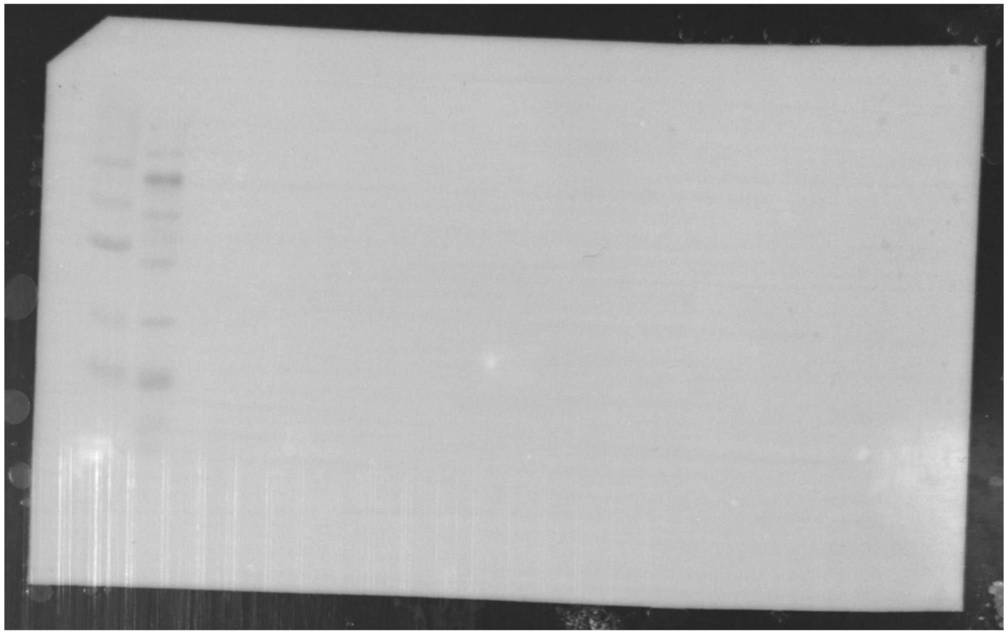
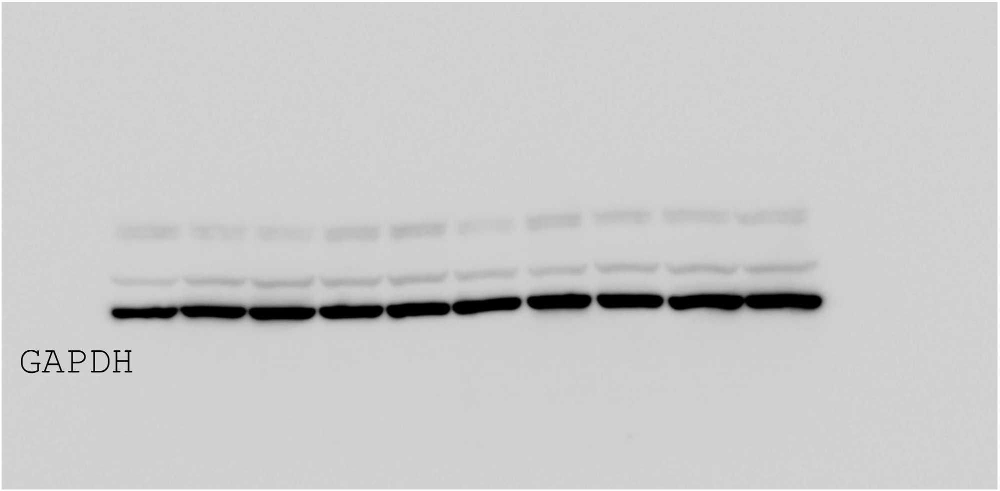
GAPDH
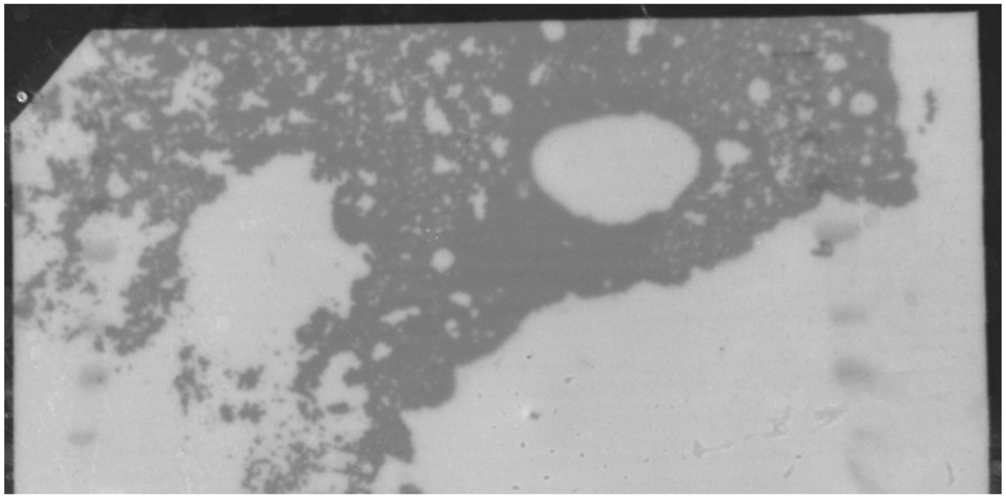

Supplement: Supplementary file 1 [file biology-14-00527-s001.zip › biology-3538612-supplementary.pdf]
